# The spectrum of neurological disorders presenting at a neurology clinic in Yaoundé, Cameroon

**DOI:** 10.11604/pamj.2013.14.148.2330

**Published:** 2013-04-04

**Authors:** Callixte Kuate Tegueu, Séraphin Nguefack, Jacques Doumbe, Yannick Fogoum Fogang, Paul Chimi Mbonda, Elie Mbonda

**Affiliations:** 1 Department of Neurology, Douala Laquintinie Hospital, Douala, Cameroon; 2 Department of Paediatric Neurology, Yaounde Gyneco-Obstetric and Paediatric Hospital, Yaoundé, Cameroon; 3 Department of Neurology, Fann Teaching Hospital, Dakar, Senegal

**Keywords:** classification, epidemiology, hospital prevalence, neurological disorders, resource poor setting, urban Africa, Cameroon

## Abstract

**Introduction:**

The burden of these neurological diseases is higher in developing countries. However, there is a paucity and scarcity of literature on neurological diseases in sub-Saharan Africa. This study was therefore undertaken to determine the pattern of neurological diseases in this setting and then, compare to those elsewhere in the African continent and also serve as a baseline for planning and care for neurological disorders in Cameroon.

**Methods:**

The study was conducted at the Clinique Bastos, in Yaoundé, city capital of Cameroon, centre region. Over a period of six years, all medical records were reviewed by a neurologist and neurological diagnoses classified according to ICD-10.

**Results:**

Out of 4526 admissions 912 patients (20.15%) were given a neurological diagnosis. The most frequent neurological disorders were headache (31.9%), epilepsy (9.86%), intervertebral disc disorder (7.67%), followed by lumbar and cervical arthrosis, polyneuropathy, stroke, Parkinson disease and dementia. According to ICD-10 classification, Episodic and paroxysmal disorders (headaches, epilepsy, cerebrovascular, sleep disorders) were observed on 424 (46.48%) patients; followed by nerve, nerve root and plexus disorders in 115 (12.6%) patients.

**Conclusion:**

The above data emphasizes that neurological disease contributes substantially to morbidity in an urban African hospital. Headaches, epilepsy and intervertebral disc disorders are major causes of morbidity.

## Introduction

Neurological disorders are responsible of more than 20% of the world's burden of disease while neurological and psychiatric disorders are responsible for up to 28% of all years of life lived with disability [1]. Neurological disorders contributed to 92 million disability-adjusted life-years (DALY) in 2005 and were projected to 103 million in 2030 [1, 2]. The burden of these neurological diseases is higher in developing countries that constitute about 85% of the world's population [1, 2]. However, there is a paucity and scarcity of literature on neurological diseases in Africa. This study was therefore undertaken to determine the pattern of neurological diseases in this setting and then, compare to those elsewhere in the African continent and also serve as a baseline for planning and care for neurological disorders in Cameroon.

## Methods

The records of all out-patients consultations from May 2005 to December 2011 were collected at the Clinique Bastos, the sole clinic with adult neurological consultation at the study period in Yaoundé, political capital of Cameroon. The following data were extracted from these patient's medical records: age, sex, profession, residence, referral person or personnel, date of first consultation, the main complaint for the 1^st^ consultation and the diagnosis. We classified neurological diseases according to the 10^th^ revision of the International classification of diseases as follows [2]: (1) Inflammatory diseases of the central nervous system, (2) Systemic atrophies primarily affecting the central nervous system, (3) Extrapyramidal and movement disorders, (4) Other degenerative diseases of the nervous system, (5) Demyelinating diseases of the central nervous system, (6) Episodic and paroxysmal disorders, (7) Nerve, nerve root and plexus disorders, (8) Polyneuropathies and other disorders of the peripheral nervous system, (9) Diseases of myoneural junction and muscles, (10) Cerebral palsy and other paralytic syndromes, (11) Other disorders of the nervous system. The diagnoses were made clinically with laboratory and radiological confirmation. The laboratory tests depended on the suspected neurological diagnosis process and included: Full blood count, ESR, serum biochemistry, serological tests, and microbiology. Lumbar puncture and muscle biopsy were used when indicated. The radiological tests were CT-scan (brain and spine), spine X-ray, rarely MRI that became available only in 2010 and because of affordability were used when it was indicated. Electrophysiological tests included: Electroencephalography (EEG), Electroneuromyography (ENMG), and Electrocardiography (ECG). Viral studies, histochemistry and other higher technology neurologic investigative tools were not used for the diagnoses of these patients because these facilities were not available.

Statistical analyses were done using the software Epi Info version 5.3.1 (CDC, August 13, 2008). Quantitative variables were presented as mean ± standard deviation, and qualitative variables as percentages.

## Results

**Prevalence of neurological disorders:** From May 2005 to December 2011, a total of 912 patients with neurological disorders were admitted within approximately six years for outpatient consultation. Not included were patients with head injuries presenting without neurological signs or symptoms and those with non-neurological backache. In the same period of time, a total of 4526 patients were admitted. Thus the hospital-based prevalence of patients with neurological diseases was 20.15%.

**Figure 1:**
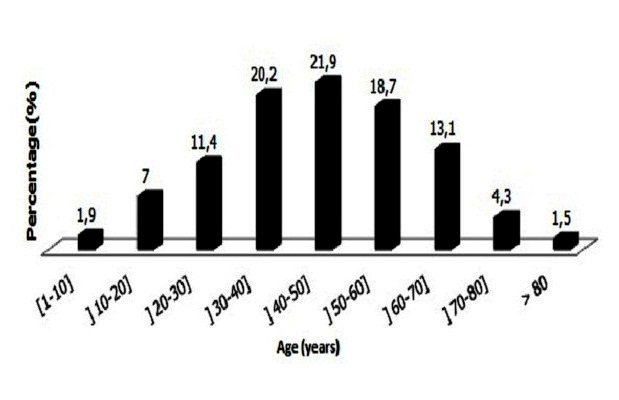
Distribution of patients by age

**Age and sex distribution:** The sex distribution of patients was 464 (50.9%) females and 448 (49.1%) males, giving a sex ratio (F:M) of 1.03:1. The ages of the patients ranges from 1 to 97 years with the mean of 44.83 ± 17.13 years and median of 45 years. The age distribution of patients is shown on Figure 1.

**Occupation:** Out of the 912 patients, 31.8% were civil servant or salaries, 17.65% traders or craftsmen, 17.54% working at home, 13.82% pupils or students, 9% retired, 8.77% of liberal profession and 1.42% were farmers.

**Place of residence:** 666 (73%) patients were living in Yaoundé, 3.9% came from neighbouring town, 19% came from other nine region of Cameroon, and 4.1% from other countries (Chad, Equatorial Guinea, Central African Republic and France).

**Source of referral:** 38.4% of patients were referred by other specialized doctors, 35.7% by general practitioners, 9.5% by a nurse, 14.4% came alone and 2% by other health professional (physiotherapist, speech therapist).

**Presenting complaint:** The main complaint on consultation was headache (28.7%), followed by lumbar pain (12%) and cervical pain (10.8%). The 10 leading complaint are presented in Table 1.

**Table 1:** The 10 leading complaint on outpatient consultation

The 10 leading complaints	Frequency	Percentage (%)
Headaches	262	28.7
Lumbar pain	110	12
Cervical pain	99	10.8
Seizures	76	8.3
Pain on limbs	75	8.2
Weakness of a limb	56	6.1
Tremor and movement disorders	42	4.6
Loss of consciousness	35	3.8
Cognitive disorders	25	2.7
Behaviour changes	24	2.6

**Neurological diagnoses:** The frequency of the various neurological diseases groups according to ICD-10 classification are showed in Table 2. Episodic and paroxysmal disorders (headaches, epilepsy, cerebrovascular, sleep disorders) were observed on 424 (46.48%) patients. The commonest diseases in this study were headaches followed by epilepsy and intervertebral disc disorders. The frequency of the ten leading neurological diseases in outpatient consultation is shown on Table 3.

**Table 2:** Neurological diseases according to ICD-10 classification

ICD-10 Classification		Frequency	Percentages (%)
Episodic and paroxysmal disorders	Headaches	291	31.9
	Epilepsy	91	9.98
	Cerebrovascular	32	3.5
	Sleep disorders	10	1.1
Nerve, nerve root and plexus disorders		115	12.6
Other disorders of the nervous system		105	11.52
Extrapyramidal and movement disorders		45	4.94
Polyneuropathies and other disorders of the peripheral nervous system		38	4.17
Inflammatory diseases of the central nervous system		22	2.42
Other degenerative diseases of the nervous system		15	1.64
Cerebral palsy and other paralytic syndromes		9	0.99
Diseases of myoneural junction and muscle		7	0.77
Systemic atrophies primarily affecting the central nervous system		3	0.32
Demyelinating diseases of the central nervous system		2	0.22
Non neurological disorders		127	13.93
**Total**		912	100

**Table 3:** The ten leading diseases in outpatient neurological consultation in Yaoundé

The ten leading diseases	Frequency	Percentages (%)
1. Headaches	291	31.9
2. Epilepsy	90	9.86
3. Intervertebral Disc Disorder	70	7.67
4. Lumbar Arthrosis	51	5.6
5. Cervical Arthrosis	45	4.93
6. Depression	39	4.27
7. Ployneuropathy	38	4.16
8. Stroke	29	3.18
9. Parkinson Disease	27	2.96
10. Dementia	26	2.85
**Total**	**706**	**77.41**

## Discussion

This study aimed at the overall description of neurological diseases in an African urban hospital. The present study supports that neurological disorders are frequent in developing countries. Of all 4526 admissions 20.15% were due to neurological disorders. Other African studies showed similar results. A recent study from two hospital sites in Ethiopia showed much higher hospital-based prevalences of 18% and 24.7% [1]. As for our study, patients’ data were collected retrospectively, which may account for the high prevalence rates compared to other studies. Osuntokun (1971) analysed neurological illnesses from 1957 - 1969 at the University College Hospital, Ibadan, Nigeria and calculated a minimum prevalence of neurological diseases in the hospital population of 4.2%, not including patients with acute head injury, traumatic paraplegia and febrile convulsions [2]. A retrospective study from Kwasa (1992) carried out in Kenya showed that neurological disorders made up for 7.5% of all medical conditions at the Kenyatta National Hospital [3]. In a Zambian hospital, Birbeck (2001) observed that 10% (186 patients) of the 1886 hospital admissions during a 13-week period were due to neurological diseases [4].

**Demographic characteristics of the sample:** The mean age of our patients was 44.83 ± 17.13 years and the sex ratio was 1.1/1. The majority of pediatric neurology patients in Yaoundé were seen in a Paediatric public hospital and only few of them came for consultation in this clinic. These findings are similar to those reported in Madagascar and Ivory Coast [3, 4]. In contrast, Winkler et al [5] in Tanzania reported a mean age of 26.4 years in a rural area and a sex ratio of 1.31 in favor of males. The majority of patients (74.1%) were referred by other doctors. At the time of the study, there were less than 10 neurologists in the countries, and only four in the capital city Yaoundé. This may be the reason why the vast majority of patients were referred by other health practitioners.

**Pattern of neurological disease:** The data from Yaoundé-Cameroon, display the same pattern of neurological diseases as those found in the majority of tropical countries. The leading position of episodic and paroxysmal disorders (46.48%) is similar to the findings by Anand et al (1993) in India [1], Chapp-Jumbo in Nigeria [5], Andrantseheno in Madagascar [6] and Winkler et al in rural Tanzania [7]. Early diagnosis and adequate treatment can prevent death in many of these cases like stroke. On the other hand many neurological illnesses are chronic, such as epilepsy, and represent a huge socioeconomic burden to the patients and their families. Early and adequate treatment may prevent chronicity or secondary damage and increase the patients’ and their families’ quality of life [7].

The top ten diseases in our study was headache (31.9%), epilepsy (9.86%), intervertebral disc disorder (7.67%), followed by lumbar and cervical arthrosis, depression, polyneuropathy, stroke, Parkinson disease and dementia. Despite the lack of population-based studies, the leading position of headache and epilepsy among our hospital-based cases confirm their importance in public health. This proves that headaches and epilepsy are a major public health problem in our neurology outpatient setting. However, this pattern is not homogenous in different tropical countries: Nigerian study reported stroke in 61.6% of cases followed by meningitis and encephalitis in 13.4% and epilepsy only on 3.4% [5]. Another study in Nigeria by Osuntokun [2] reported infections of the nervous system as the commonest neurological problem, followed by vascular disease and epilepsy. Indian studies reported a high prevalence of epilepsy in 20.6% [8] while in Ivory Coast where all the patients were admitted for hospitalization, stroke was reported in 42.18% followed by cerebral toxoplasmosis (17.9%) and meningo-encephalitis in 11.9% [9]. In Madagascar, the most prevalent disease was epilepsy (28.75%) followed by chronic headache (20.95%), peripheral neuropathies (13.75%) and stroke in 11.3% [6]. In Ethiopia [10], cerebrovascular disease was the most common neurological diseases seen (45%), the second commonest disorder been bacterial meningitis (12%). In Nairobi-Kenya [3], the 3 commonest diseases were meningitis (23.1%), epilepsy (16.6%) and cerebrovascular diseases (15.0%).The classification of neurological diseases in urban Africa is important in terms of diagnostic approach and therapy as well as the prognosis. Diagnoses belonging to top ten diseases (Table 3) are important to recognize as early as possible in the diagnosis process as often there are crucial therapeutic implications. It is important that standard treatment regimes, often Ministry of Public Health based, are adhered to these diseases. Recognition of diseases belonging to this group ideally leads to immediate life-saving treatment.

Concerning chronic headaches, post graduate training is needed to improve the diagnosis and management of the most frequent form, the tension-type headache. Drugs availability and cost must be taken into account in case of long term treatment; patient's compliance depending on these extramedical factors [6]. Traditional pharmacopoeia and alternative medicine are optional issues for developing countries, but their use must be regulated.

Peripheral neuropathies are probably underrated, due to hospital recruitment; the reasons may be a self management with vitamin B tablets, and difficulty for doctors to make an accurate diagnosis. The endowment of essential facilities like ENMG, nerve conduction measurement, will enrich the knowledge about these diseases, common in tropical countries; where certain aetiologies like diabetes, HIV-infection and alcohol consumption are frequently encountered. The surprising high rate of intervertebral disc disorder as well as cervical and lumbar arthrosis results likely on local inadequacy in professional training: indeed, almost 35% of the patients were traders, craftsman or working at home, and naive load handlers are recruited without any technical skill learning. However, lumbar disk prolapse is considered as a rather rare condition in Africans [2].

Although the proportion of our stroke cases represent only 3.18% of cases, compared to those reported in the majority of African hospital-based studies [2, 3, 5, 9–11], they constitute a real human and economic burden; the lack of appropriate diagnosis and urgent therapeutic supplies may explain the high mortality rate, and the worse functional prognosis [6].

## Conclusion

The patterns of neurological diseases in Yaoundé (Cameroon) are globally similar to that of the majority of tropical countries. Headache, epilepsy and intervertebral disc disorders are the commonness diseases encountered while stroke and degenerative diseases like Parkinson diseases are rare. An epidemiological survey on general population and epidemiological research on risk factors are important to build up an appropriate preventive and curative policy
